# Medication adherence framework: A population‐based pharmacokinetic approach and its application in antimalarial treatment assessments

**DOI:** 10.1002/psp4.13119

**Published:** 2024-03-25

**Authors:** Junjie Ding, Richard M. Hoglund, Joel Tarning

**Affiliations:** ^1^ Mahidol Oxford Tropical Medicine Research Unit Mahidol University Bangkok Thailand; ^2^ Centre for Tropical Medicine and Global Health, Nuffield Department of Clinical Medicine University of Oxford Oxford UK; ^3^ The WorldWide Antimalarial Resistance Network Oxford UK

## Abstract

We reported here on the development of a pharmacometric framework to assess patient adherence, by using two population‐based approaches – the percentile and the Bayesian method. Three different dosing strategies were investigated in patients prescribed a total of three doses; (1) non‐observed therapy, (2) directly observed administration of the first dose, and (3) directly observed administration of the first two doses. The percentile approach used population‐based simulations to derive optimal concentration percentile cutoff values from the distribution of simulated drug concentrations at a specific time. This was done for each adherence scenario and compared to full adherence. The Bayesian approach calculated the posterior probability of each adherence scenario at a given drug concentration. The predictive performance (i.e., Youden index, receiver operating characteristic [ROC] curve) of both approaches were highly influenced by sample collection time (early was better) and interindividual variability (smaller was better). The complexity of the structural model and the half‐life had a minimal impact on the predictive performance of these methods. The impact of the assay limitation (LOQ) on the predictive performance was relatively small if the fraction of LOQ data was less than 20%. Overall, the percentile method performed similar or better for adherence predictions compared to the Bayesian approach, with the latter showing slightly better results when investigating the adherence to the last dose only. The percentile approach showed acceptable adherence predictions (area under ROC curve > 0.74) when sampling the antimalarial drugs piperaquine at day 7 postdose and lumefantrine at day 3 postdose (i.e., 12 h after the last dose). This could be a highly useful approach when evaluating programmatic implementations of preventive and curative antimalarial treatment programs in endemic areas.

**Study Highlights**

**WHAT IS THE CURRENT KNOWLEDGE ON THE TOPIC?**

Adherence to medication is critical to the outcome of the treatment. To date, many approaches have been proposed for adherence assessment, such as patient self‐reports, pill counts, etc. However, pharmacology‐based approaches, with the measurement of drug concentrations, are considered to be the most objective approach for adherence assessment of recent dose events.

**WHAT QUESTION DID THIS STUDY ADDRESS?**

The study assessed the predictive performance of two population‐based pharmacokinetic (PK) approaches (i.e., percentile approach and Bayesian approach) for three consecutive dose events. The predictive performance of both approaches was highly affected by sample collection time and between‐patient PK variability. However, the performance was not influenced by the complexity of model structure or the half‐life of the drug. The percentile approach was used to successfully derive concentration cutoff values for two of the most commonly used partner drugs in antimalarial therapy.

**WHAT DOES THIS STUDY ADD TO OUR KNOWLEDGE?**

The study provides a pharmacometric model‐based framework to quantitatively assess medication adherence for recent dose events.

**HOW MIGHT THIS CHANGE DRUG DISCOVERY, DEVELOPMENT, AND/OR THERAPEUTICS?**

The developed framework might be a highly useful and complementary tool to accurately assess medication adherence in a clinical setting.

## INTRODUCTION

Adherence to medication is crucial to treatment outcome and has recently received an increased interest in the scientific community.^1^, ^2^ Poor adherence could result in suboptimal concentrations, thereby increasing the risk of treatment failures.^3^ When treating infectious diseases, suboptimal treatment can also lead to increased transmission and resistance development. To date, a number of direct and indirect methods has been developed to investigate treatment adherence in patients. Indirect methods include patient questionnaires, patients' self‐reports, pill counts, rates of prescription refills, assessment of the clinical response, and electronic medication monitors. Self‐reports and household survey questionnaires are more feasible and available at a relatively low cost, and are the most commonly used evaluations techniques today.^4^ However, due to recall bias by patients, these methods often result in an overestimated adherence.^3^ The situation might be even more challenging in developing countries as patients might be remote to the study facility and hesitant to complete a recall visit, resulting in additional barriers for conducting clinical trials in developing countries.^5^ In contrast, customized electronic monitoring devices are more precise and makes it easy to quantify the adherence.^6^ However, these devices are expensive, limiting their use in routine and large clinical trial settings, and especially in resource‐limited countries.

Another method to measure adherence is the pharmacokinetic (PK)‐based approaches, in which levels of drug and/or its metabolites are measured to assess the adherence. These methods are considered to be objective, and has recently achieved increasing interest in research.^7^, ^8^, ^9^, ^10^ In theory, the distribution of drug concentrations at a given sampling time should be different in patients with full adherence compared to patients that did not take medications as instructed. Based on this assumption it should be possible to distinguish full and poor adherence by comparing their measured concentrations at a given time. With the help of population‐based PK modeling and simulation, the variability between patients as well as the distribution in drug concentrations can be quantified. To the best of our knowledge, only two approaches based on population PK modeling have been developed to access medication adherence (i.e., the percentile method^10^ and the Bayesian method).^7^ In the reported percentile method, a measured concentration is compared with a specific percentile cutoff value (e.g., 1%, 2.5%, or 5%) based on the distribution of concentrations associated with full adherence.^10^ The Bayesian approach calculates the posterior probability of a given concentration for each dosing scenario (e.g., missing one dose) based on the prior probability and conditional probability (probability of a specific concentration to occur under a specific condition, i.e., a full adherence or nonadherence scenario).^7^ However, the performance (e.g., sensitivity and specificity) for adherence predictions and the impact of interindividual variability (IIV) in PK models have not been investigated for these methods.

Malaria is a mosquito‐borne parasitic disease. According to the World Health Organization (WHO), there were an estimated 247 million malaria cases and 619,000 malaria deaths in 2021.^11^ Artemisinin‐based combination therapies (ACTs) are the WHO‐recommended first‐line treatment for malaria.^12^ Poor adherence to antimalaria therapy has been shown to lead to lower drug concentrations, resulting in a high risk of therapeutic failure (i.e., recurrent malaria infection).^13^ Low drug levels can also contribute to the development and spread of drug‐resistant parasites. Regarding the adherence to antimalarial therapy, a systematic review based on 55 clinical trials summarized the different adherence assessment approaches in patients receiving ACTs for malaria treatment, in which 47 clinical trials targeted *P. falciparum*, five trials targeted *P. vivax* treatment, and three trials evaluated both malaria species.^4^ The studies reported a very wide range of adherence results across different studies and settings, ranging from 17%–98% for *P. vivax* and 1.5%–100% for *P. falciparum*. Several studies demonstrated that measured drug concentrations were lower in nonadherent patients with malaria treated with lumefantrine or amodiaquine compared to patients with full adherence.^14^, ^15^, ^16^, ^17^, ^18^, ^19^ Some malaria clinical trials proposed PK‐based approaches and used concentration cutoff values for adherence assessments, such as assay limit of detection on day 3^20^ and day 7 after the first dose^21^ or at the time of recurrent malaria.^22^ Another trial used the lower boundary of the 95% confidence interval of observed concentrations collected on day 2.^23^ Nevertheless, the predictive performance of these concentration cutoff values has not been systemically evaluated. Adherence assessment of antimalarial therapy, using population PK‐based approaches, has not been reported before. Large pooled pharmacometric evaluations of piperaquine^24^ and lumefantrine,^25^ based on pooled individual level participant data, has been reported allowing precise and accurate characterization of the PK properties and IIV of these drugs. Therefore, these two drugs were used as model drugs to evaluate the clinical application of the two adherence assessment approaches.

In this study, we investigated the predictive performance of two population PK‐based approaches (i.e., the percentile and the Bayesian method) to assess drug adherence. Furthermore, both modeling approaches were applied to evaluate antimalarial drug adherence to two of the most commonly used partner drugs, piperaquine and lumefantrine.

## METHODS

### Dosage regimen and adherence scenarios

In malaria treatment, the first dose is commonly observed. As the dosing scenario was based on a 3‐day daily dose, here, we included all possible nonadherence scenarios for completion and to be able to generalize our results to other diseases and treatment settings.

**FIGURE 1:**
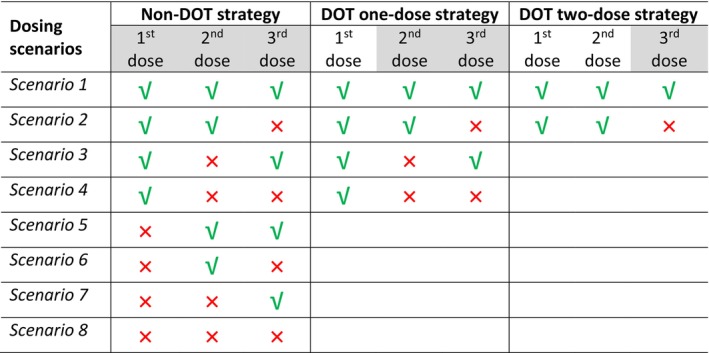
Available simulation scenarios in terms of different levels of directly observed therapy (DOT); all the doses were taken without observation (non‐DOT strategy); the first dose was directly observed (DOT one‐dose strategy); and the first and second dose was directly observed (DOT two‐dose strategy). The shaded scenarios represent the dose events for adherence assessments. The patients were assumed to a take daily dose of 200 mg for three consecutive days for full adherence. The tick and cross symbols represent patients taking or missing the scheduled dose.

Patients were assumed to be administered a daily dose of 200 mg for 3 days (a total of 3 dose events) for full adherence, based on the most commonly used dose regimen in the treatment of uncomplicated malaria. However, this is not exclusive for malaria but used as an illustrative example of the developed methodology. Nonadherence was evaluated for each of the three possible dosing strategies. Nonadherence was simulated assuming three distinctly different dosing strategies: (1) all the doses were taken without observation (non‐directly observed therapy [DOT] strategy) and the adherence to all three doses were assessed; (2) the first dose was directly observed (DOT one‐dose strategy), and the adherence to the second and third dose was assessed; (3) the first and second dose was directly observed (DOT 2‐dose strategy) and the adherence of the third dose was assessed. For the above three strategies, there are eight, four, and two possible combinations of dosing events (simulation scenarios), respectively (Figure 1). The probability of each nonadherence scenario and event may vary based on disease and treatment setting, and also patient characteristics.^26^ The prevalence of each nonadherence scenario is rarely reported. Here, we assumed equiprobable prevalence for each scenario.

### Definition of complete and poor adherence

Full adherence was defined as correctly taking a daily dose for a total of 3 days as instructed, whereas the patients missing at least one of the three doses was categorized as having poor adherence.

### Population PK model used in the simulation

A drug with first‐order absorption and one‐compartment disposition kinetics was used as the basis for the theoretical simulation study. PK parameters and their IIV, as well as the unexplained residual error (RUV) are shown in Table 1. The IIV and RUV were assumed to be normally distributed with a zero mean and variance *ω* ^2^ and *σ* ^2^, respectively. IIVs (presented as coefficient of variation [%CV]) of 20%, 40%, and 60% were investigated, representing a small, medium, and large magnitude of variability, respectively. The median time to reach maximum concentration and half‐life were 4.3 and 23.1 h, respectively.

**TABLE 1:** Pharmacokinetic parameters used in simulation.

Parameters	One‐compartment model		Two‐compartment model		Three‐compartment model		One‐compartment model with a shorter half‐life	
	Typical value	CV% of IIV	Typical value	CV% of IIV	Typical value	CV% of IIV	Typical value	CV% of IIV
Ka (/h)	0.5	20, 40, 60	0.5	40	0.5	40	0.5	40
F (%)	100	20, 40, 60	100	40	100	40	100	40
CL/F (L/h)	3	20, 40, 60	10	40	2.5	40	2.15	40
Vc/F (L)	100	20, 40, 60	100	40	10	40	25	40
Q1/F (L/h)	–	–	5	40	2	40	–	–
Vp1/F (L)	–	–	100	40	25	40	–	–
Q2/F (L/h)	–	–	–	–	1.2	40	–	–
Vp2/F (L)	–	–	–	–	20	40	–	–
Proportional RUV (variance)	0.04	–	0.04	–	0.04	–	0.04	–
Half‐life (h)	23		24		24		8	

*Note*: CV% is calculated as $\sqrt{\omega^{2}}$.

Abbreviations: CL/F, apparent elimination clearance; CV%, coefficient of variation; F, relative bioavailability; IIV, interindividual variability; Ka, first‐order absorption constant; Q/F, apparent inter‐compartmental clearance; RUV, residual error; Vc/F, apparent volume of central compartment; Vp/F, apparent volume of peripheral compartment.

### Software

Stochastic simulations were conducted using the deSolve package and post processing of output was undertaken in R software (version 3.2.3, www.r‐project.com).

### Simulations

Stochastic simulations (2000 individuals) were performed for each of all possible scenarios of dosing events to represent the population characteristics, based on the population PK parameters in Table 1. Continuous dense plasma‐concentration time curves (concentrations sampled every 1 h) were simulated for up to three half‐lives (72 h) after the last dosing. We evaluated the adherence at various PK timepoints, from 1 h up to 72 h after the last dosing.

### Description of the percentile method

**FIGURE 2:**
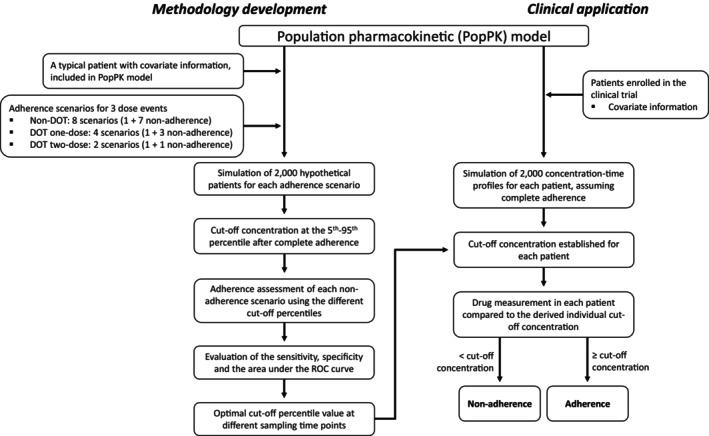
Flowchart of developed adherence methodology and clinical application using the percentile approach. DOT, directly observed therapy.

Briefly, the cutoff concentration at a given percentile value (e.g., 5%) were calculated at a given time *t*, based on 2000 simulated individuals with full adherence to the treatment.^10^ The nonadherence scenarios were simulated and individuals with concentrations at time *t* below that given cutoff value derived from full adherence simulation, were assigned to be poor adherence individuals. Different cutoff percentiles, from 5% to 95% (5%‐unit increments) in the full adherence scenario, were investigated as optimal cutoff values in the nonadherence assessment. The assessment of optimal cutoff percentiles was based on the degree of false negative rate (specificity 5%–95%). The flow chart of methodology development of the percentile approach is shown in Figure 2.

### Description of the Bayesian approach

The Bayesian approach, which incorporate both a prior probability and a conditional probability, developed by Barriere et al.,^7^ was used to assess the medication adherence. In brief, all individual concentrations at time *t* from all scenarios were pooled together and 20 concentration bins from less than 5% to greater than 95% percentiles (5%‐unit increments) were generated, based on the pooled concentrations distribution. The conditional probability of a specific concentration for a given scenario could be calculated in each of these 20 bins. Incorporation of the conditional probability and the assumption of equiprobable prior probability, the posterior probability for each scenario at a given concentration could be calculated with the Bayesian formula (Equations 1 and 2). The most plausible scenario of a given concentration at time *t*, is the one with the largest posterior probability among all evaluated scenarios.

(1) $$P\left(\omega_{j}|C\right)=\frac{P\left(\omega_{j}\right)\cdot p\left(C|\omega_{j}\right)}{p\left(C\right)}$$

(2) $$p\left(C\right)=\sum_{j}P\left(\omega_{j}\right)\cdot p\left(C|\omega_{j}\right)$$

where, *C* is a given concentration, $p\left(C\right)$ is the full probability, $P\left(\omega_{j}\right)$ is the prior distribution of each adherence scenario, with the assumption of equiprobable probability for each adherence scenario (i.e., 0.5, 0.25, and 0.125 for each scenario in DOT two‐dose, DOT one‐dose and non‐DOT conditions, respectively). $p\left(C|\omega_{j}\right)$ is the conditional probability of a concentration at a given scenario, and $P\left(\omega_{j}|C\right)$ is the posterior probability of a scenario for a given concentration.

### Predictive performance of two PK‐based approaches for the adherence assessment

The predictive performance was measured by assessing sensitivity, specificity, Youden's index, as well as the receiver operating characteristic (ROC) curve. A two‐by‐two table was used to calculate the sensitivity and the specificity for both the Bayesian and the percentile method. For both methods, sensitivity is the proportion of patients with a true poor adherence who was also assigned to have poor adherence, and specificity is the proportion of patients with full adherence who was also predicted to have full adherence, as illustrated in a two‐by‐two table in Table S1. Youden's index, defined as the sum of sensitivity and specificity minus 1, was used to access the overall predictive performance.^27^ A higher Youden's index indicates better predictive performance in predicting poor adherence.

The ROC graphs were constructed by plotting sensitivity versus one‐specificity. The area under the ROC curve (AUC) was calculated with the trapezoidal method, in order to assess the overall predictive performance. In general, an AUC of less than or equal to 0.5 suggests no predictive ability, 0.7–0.8 is considered acceptable or fair predictive performance, 0.8–0.9 is considered excellent predictive performance, and greater than or equal to 0.9 is considered outstanding predictive performance,^28^ for discrimination between the full and poor adherence. An AUC greater than 0.75 is usually recommended for clinical decision purposes.^29^ It is used along with sensitivity (probability of true poor adherence) and specificity (probability of true full adherence) parameters to interpret the predictive performance of the approach.

### The impact of IIV

The impact of IIV on the predictive performance of both the percentile method and the Bayesian approach were investigated using the DOT one‐dose strategy, whereas the second and third doses were not observed. The sensitivity, specificity, as well as the Youden's index were evaluated for different magnitudes of IIV for all the PK parameters (%CV of IIV: 20%, 40%, and 60%, respectively).

### The impact of more complicated structure models

The basic one‐compartment disposition model was extended to a more advanced two‐ and three‐compartment disposition model, and the predictive performance of both the percentile and the Bayesian method were evaluated. The results were compared with those derived from the one‐compartment disposition model. Parameters for the two‐compartment model were chosen to result in a similar terminal elimination half‐life of ~24 h (Table 1).

### The impact of half‐life

The impact of half‐life was investigated on the predictive performance of both the percentile and the Bayesian method. Parameters for the one‐compartment model were chosen to reflect a terminal elimination half‐life of ~8 h, as shown in Table 1. The results were then compared with those from a drug with a half‐life of 24 h.

### Impact of limit of quantification

The impact of a truncated concentration distribution (i.e., data censoring due to bioanalytical assay limitations) was investigated on the predictive performance of the percentile method at a given timepoint (24 h, 1 half‐life) for the DOT one‐dose strategy. Concentration percentiles at this specific timepoint were derived from simulating full adherence, but with different levels of assay limit of quantification (LOQ) limits, resulting in between 0% and 50% of data below the LOQ. Key parameters regarding the predictive performance, such as sensitivity, specificity, and AUC under the ROC curve were evaluated.

### Application of the adherence methodology in antimalarial drug therapy

The adherence methodology was also applied using previously reported PK models for the antimalarial drugs piperaquine and lumefantrine, according to the overall procedures suggested in Figure 2.

A large‐scale pooled PK analysis of piperaquine, combining 11 clinical trials (8776 samples from 728 individuals) in healthy volunteers (*n* = 50) and patients with uncomplicated malaria (*n* = 678), showed that piperaquine PK properties were described accurately by a three‐compartment disposition model with transit absorption.^24^ A total of 301 children below the age of 5 years was included in the analysis, and the age to reach 50% of full maturation of the elimination clearance (MF_50_) was 0.575 years, and the Hill coefficient in the maturation function was 5.11. This resulted in 95% of the full elimination clearance (MF_95_) at 1 year of age, resulting in a negligible age effect on clearance in patients greater than 1 year. The relative bioavailability was increased by 23.7% between each dosing occasion in patients with malaria infection.

The PK properties of lumefantrine has also been evaluated in a large pooled analysis based on 4122 patients from 26 different studies in adults (*n* = 2665), pregnant women (*n* = 123), and children below 10 years old (*n* = 1457) with uncomplicated *P. falciparum* malaria. This study found that lumefantrine were described accurately by a two‐compartment disposition model with first‐order absorption.^25^ Lumefantrine exposure decreased with increasing pretreatment parasitemia, and showed dose‐dependent saturation of the absorption. Moreover, pregnancy status increased the absorption rate of lumefantrine by 35.2%.

PK parameters and variability presented in the published papers^24^, ^25^ were used for the current simulations. All the patients were assumed to receive a daily dose of piperaquine or a twice‐daily dose of lumefantrine for a total of 3 days, following two plausible clinical dosing strategies; directly observed administration of the first dose only (DOT first‐dose) followed by non‐observed therapy for the remaining doses; and non‐observed therapy of all doses (non‐DOT). The dosage of piperaquine and lumefantrine were chosen based on bodyweight as given by the WHO malaria treatment guidelines.^12^ The simulation was based on the covariate‐parameter relationships in previous pooled PK model in patients with malaria infection. With the individual covariate data, we were able to simulate PK concentrations in all conditions for piperaquine (bodyweight and age) and lumefantrine (bodyweight, pretreatment parasitemia, dose, and pregnant status). We reported cutoff concentrations for each kg bodyweight in patients weighting more than 11 kg receiving piperaquine. For younger children (bodyweight <11 kg) receiving piperaquine, and all patients receiving lumefantrine, we used an R script (Appendix S1) to simulate and derive cutoff concentrations for different combinations of covariates.

The plasma concentrations of piperaquine and lumefantrine on day 7 post first‐dose has been reported to be a biomarker for therapeutic response,^30^, ^31^ with suggested target lumefantrine concentrations of 175 ng/mL,^32^ and a target piperaquine concentration of 30 ng/mL.^33^, ^34^ Day 7 concentration was therefore used for adherence assessments. In addition, the overall predictive performance was evaluated when using simulated plasma concentrations on day 3, 7, 14, and 21 instead. The WHO recommends that therapeutic outcomes should be assessed on day 28, or day 42 for drugs with longer elimination half‐lives (e.g., piperaquine). This would have been the ideal sampling time for adherence assessments, but days 28 and 42 had a low predictive performance for the investigated drugs (i.e., piperaquine and lumefantrine) and was not evaluated further in this study.

Two thousand virtual individuals were simulated for full adherence and each nonadherence scenario, based on a typical non‐pregnant patient with a median bodyweight according to that reported in the modeled populations. Both the percentile and Bayesian approach were evaluated (in the same manner as stated above). However, the Bayesian method was not evaluated for lumefantrine due too many possible dose combinations (a total of 64 simulated full and nonadherence scenarios for 6 different dosing events) making this approach unpractical.

## RESULTS

### Predictive performance – Percentile versus Bayesian approach

**FIGURE 3:**
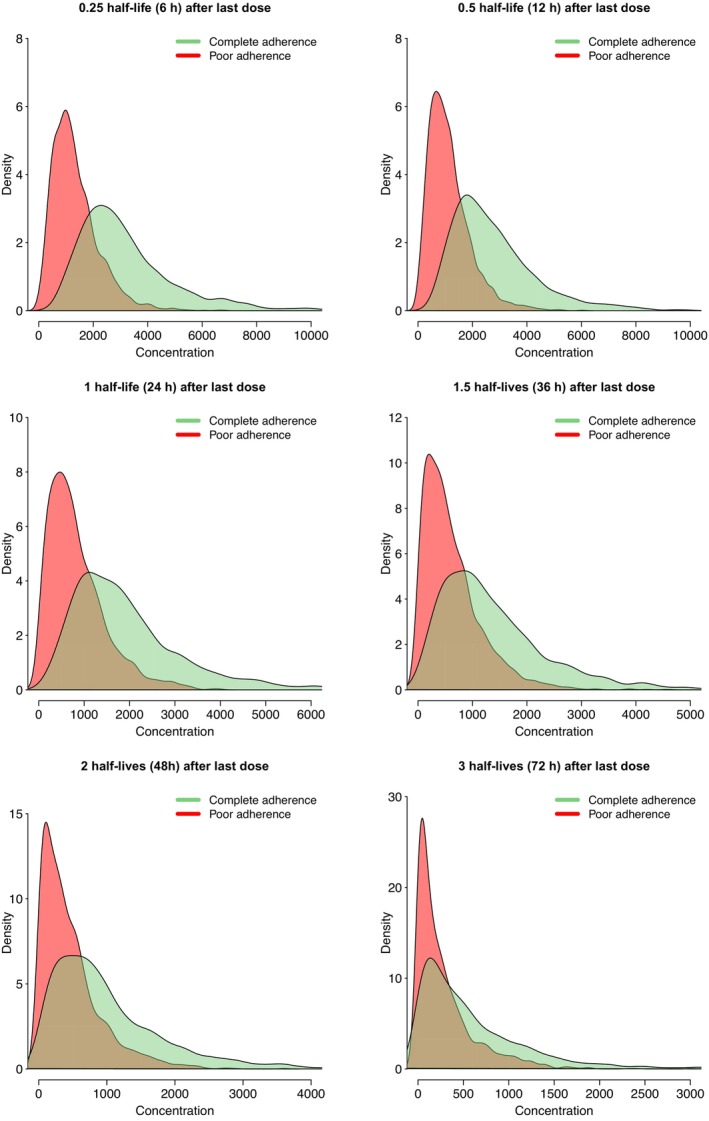
The probability density functions of drug concentration samples taken at increasing times after the last dose, when investigation the adherence to the last dose (DOT two‐dose). Red shaded curves show complete adherence and green shaded curves show poor adherence. A total of 2000 virtual individuals were simulated for complete and poor adherence in each scenario.

For the percentile method, the possibility to distinguish between full and poor adherence decreases with increasing time after dose, with an almost completely overlap between concentration distributions at three half‐lives after the last dose. The probability density of drug concentrations at different times after dose, for full adherence compared to poor adherence to the last dose (DOT two‐dose) are shown in Figure 3. The same trend could be seen when assessing adherence to the last two doses (DOT one‐dose) and non‐DOT strategies (Figure 3).

**FIGURE 4:**
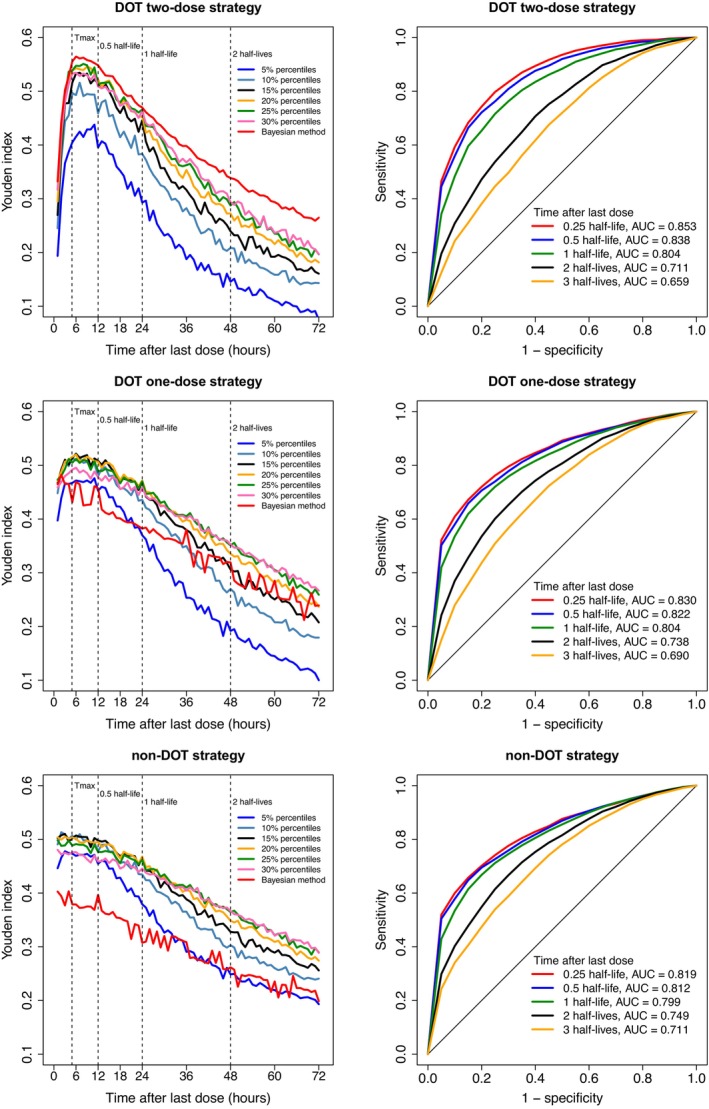
The Youden's index (left column) of the percentile method and the Bayesian approach and receiver operating characteristic (ROC) curve (right column) derived for the percentile method at different sampling times. The different adherence scenarios were evaluated in comparison to patients with complete adherence of receiving a daily dose for 3 days. DOT, directly observed therapy. The Youden index was derived for each time point (every 1 h) up to 72 h after the last dose. AUC, area under the curve; *T*_max_, time to maximum concentration.

This was further supported by the derived Youdex's, resulting in a decreasing Youdex's index with increasing time after dose (Figure 4). The same trend was seen for AUC of the ROC curve, which was also reduced with increasing time after dose. The AUCs were greater than 0.80 in all dosing scenarios when the sample was taken within one half‐life after the last dose, indicating good predictive performance for assessing adherence. If the sample was collected later than one half‐life after the last dose, the AUC was smaller than 0.75.

The optimal percentile cutoff value changed with different sampling times (Figure 4). The best Youden's index was achieved using the 15%–20% percentile for samples taken within 0.5 half‐life after last dose, the 20%–25% percentile for samples taken within 0.5–1 half‐life, and the 25%–30% percentile for samples taken after one half‐life. A similar trend was also found when evaluating adherence under non‐DOT criteria.

Overall, the percentile method performed similar or better for adherence predictions than the Bayesian approach. The predictive performance of the Bayesian approach was slightly better than the percentile method when investigating adherence to the last dose only (Figure 4).

### Predictive performance – Impact of interindividual variability

The predictive performance of both the percentile and Bayesian approach decreased with increasing IIV (Figure S1). When the magnitude of IIV was small (20%), the percentile method showed good predictive ability with an area under the ROC curve of more than 0.8, even with sampling times up to three half‐lives after the last dose. However, if the IIVs were large (60%), the predictive ability was acceptable only for sampling times within one half‐life of the last dose (AUC > 0.7), and would be clinical useless if the samples were collected later than two half‐lives after the last dose. In addition, it was found that a lower percentile cutoff value would be preferred (e.g., 10%–15% at 1 half‐life; Figure S1) in the event of low magnitude of IIV, whereas a higher percentile cutoff value was appropriate for higher magnitude of IIV (e.g., 20%–30% at 1 half‐life; Figure S1).

### Predictive performance – Impact of structural disposition model

Similar findings were shown when two‐ and three‐compartment disposition model was used instead of a one‐compartment model (Figures S2 and S3). That is, the Youden's index as well as the AUC of the ROC curve declined when the time for the sample collection increased. In order to achieve a high predictive performance for the adherence assessment (AUC > 0.80), samples should be collected within one half‐life after the last dose.

### Predictive performance – Impact of half‐life

Similar findings were seen when evaluating a model drug with a shorter half‐life (8 h) compared to a drug with a longer half‐life of 24 h (Figure S4). The Youden's index as well as the AUC of the ROC curve declined with increasing sampling time after dose. A high predictive performance for the adherence assessment (AUC > 0.80) was seen in samples being collected within one half‐life after the last dose.

### Predictive performance – Impact of LOQ

According to the distribution of simulated concentrations at one half‐life after dose, we derived the optimal percentile for adherence assessment when using different fractions of censored data (0%, 10%, 20%, 30%, 40%, and 50%). AUC under the ROC curve decreased with an increasing fraction of LOQ data (Figure S5). However, the impact of LOQ on the predictive performance was relatively small if the fraction of LOQ data was less than 20%.

### Application to antimalarial therapy – Piperaquine adherence

According to the analyses above, the percentile method was superior to the Bayesian approach when investigating the adherence of “DOT first‐dose” and non‐DOT strategies after a 3‐day dose regimen, and was therefore used in the piperaquine adherence assessment.

**FIGURE 5:**
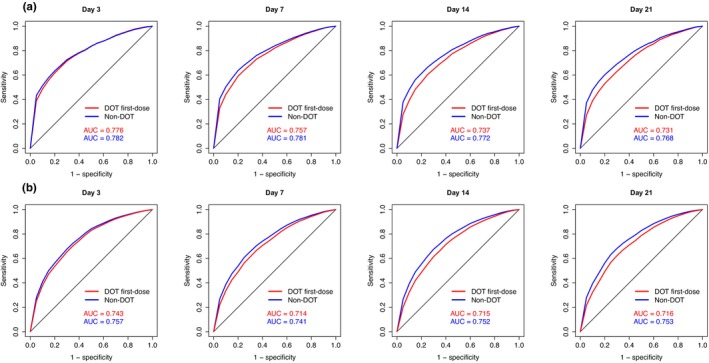
The receiver operating characteristic (ROC) curve of piperaquine (a) and lumefantrine (b) concentrations sampled at different collection times for assessment of adherence. Piperaquine was administered once daily for 3 days (a total of 3 doses). Under DOT one‐dose and non‐DOT strategies, the adherence of the last two doses and all three doses were investigated. AUC, area under the curve.

The AUC of the ROC curve for samples collected on days 3 and 7 after the first dose (which is on day 0) resulted in values greater than 0.75 under non‐DOT strategies, and should be considered clinically useful for adherence assessments (Figure 5a). When investigating adherence of the DOT first‐dose strategy, a clinically useful ROC curve result (AUC > 0.75) was seen only for day 3 and day 7 samples. Day 3 concentrations showed a higher AUC for the ROC curve than those taken at other timepoints, which is in line with the findings above that early sampling has higher predictive performance.

For day 7 concentration, the 20% percentile cutoff value (specificity of 80%) showed the highest predictive performance with a Youden's index of 0.391. The sensitivity of using the day 7 concentration and the 20% percentile cutoff were 0.637 and 0.591 for assessing adherence of DOT first‐dose and non‐DOT strategies, respectively. The full details of the two‐by‐two tables can be viewed in Table 2. That means there was a 63.7% and 59.1% probability of patients having poor adherence, respectively, if the observed day 7 concentration was below the assigned cutoff value. The probability of patients that have taken the doses correctly was 80%, when the concentration is greater than the cutoff value.

**TABLE 2:** Two‐by‐two table to derive diagnostic parameters for the adherence assessment for piperaquine and lumefantrine.

	Optimal percentile	TP (*n*)	FN (*n*)	TN (*n*)	FP (*n*)	Sensitivity (%)	Specificity (%)
Piperaquine – DOT first‐dose strategy							
Day 3	5	2335	3665	1900	100	38.9	95
Day 7	20	3546	2454	1600	400	59.1	80
Day 14	45	4637	1363	1100	900	77.3	55
Day 21	55	4989	1011	900	1100	83.2	45
Piperaquine – Non‐DOT strategy							
Day 3	5	6058	7942	1900	100	43.3	95
Day 7	20	8915	5085	1600	400	63.7	80
Day 14	45	11,294	2706	1100	900	80.7	55
Day 21	55	11,971	2029	900	1100	85.5	45
Lumefantrine – DOT first‐dose strategy							
Day 3	30	40,495	21,505	1400	600	65.3	70
Day 7	35	41,252	20,748	1400	600	66.5	65
Day 14	35	41,044	20,956	1400	600	66.2	65
Day 21	35	41,556	20,444	1400	600	67.0	65
Lumefantrine – Non‐DOT strategy							
Day 3	30	85,222	40,778	1400	600	67.6	70
Day 7	30	82,695	43,305	1400	600	65.6	70
Day 14	30	85,107	40,893	1400	600	67.5	70
Day 21	30	85,915	40,085	1400	600	68.2	70

*Note*: Sensitive = TP/(TP + FN), Specificity = TN/(TN + FP).

Abbreviations: DOT, directly observed therapy; FN, false‐negative; FP, false‐positive; TN, true‐negative; TP, true‐positive.

The day 7 cutoff concentrations for patients between 11 and 100 kg are presented in Table 3, based on stochastic simulations (*n* = 2000). For patients with bodyweights under 10 kg (~1 year of age),^34^ there are an age‐related maturation of apparent elimination clearance, and also a high number of possible age‐weight combinations and, therefore, not presented here. However, day 7 cutoff concentrations for any given age and body weight can be obtained from the developed R script (Appendix S1).

**TABLE 3:** Piperaquine day 7 concentration cutoff value in patients with malaria for adherence assessment.

Bodyweight	Cutoff conc.	Bodyweight	Cutoff conc.	Bodyweight	Cutoff conc.	Bodyweight	Cutoff conc.
(kg)	(ng/mL)	(kg)	(ng/mL)	(kg)	(ng/mL)	(kg)	(ng/mL)
11	20.21	34	16.24	57	16.00	79	16.67
12	18.84	35	15.86	58	15.77	80	20.65
13	17.67	36	23.26	59	15.56	81	20.47
14	16.65	37	22.72	60	20.51	82	20.29
15	15.75	38	22.22	61	20.28	83	20.12
16	14.95	39	21.75	62	20.06	84	19.92
17	21.30	40	21.29	63	19.84	85	19.72
18	20.33	41	20.86	64	19.61	86	19.56
19	19.44	42	20.45	65	19.39	87	19.40
20	18.63	43	20.06	66	19.15	88	19.21
21	17.90	44	19.71	67	18.91	89	19.02
22	17.25	45	19.40	68	18.68	90	18.85
23	16.65	46	19.11	69	18.46	91	18.71
24	16.09	47	18.76	70	18.25	92	18.57
25	20.79	48	18.42	71	18.04	93	18.42
26	20.14	49	18.11	72	17.84	94	18.27
27	19.55	50	17.80	73	17.64	95	18.11
28	18.98	51	17.50	74	17.48	96	17.96
29	18.44	52	17.20	75	17.32	97	17.83
30	17.92	53	16.98	76	17.16	98	17.70
31	17.49	54	16.72	77	17.01	99	17.56
32	17.06	55	16.46	78	16.83	100	17.43
33	16.64	56	16.24				

*Note*: The population‐based percentile method was used to derive optimal day 7 concentration cutoff value (i.e., 20% percentile) from 2000 simulated full adherent individuals in each bodyweight band.

### Application to antimalarial therapy – Lumefantrine adherence

The AUC of the ROC curve of samples collected on days 3, 7, 14, and 21 after the first dose (on day 0) were ~0.750 for the percentile method when assessing adherence under the non‐DOT strategy, and were considered clinically useful for adherence assessment (Figure 5b). When investigating adherence of the DOT first‐dose strategy, a clinically useful ROC curve result (AUC > 0.75) was only seen for day 3 samples (i.e., 12 h after the last dose of the 6‐dose course of lumefantrine). The ROC curve results for samples collected at days 7, 14, and 21 were below 0.75 when investigating adherence under the DOT first‐dose strategy, suggesting that these cutoffs would not be clinically useful.

For day 3 samples, the 30% percentile cutoff had the highest predictive performance with a Youden's index of 0.353 and 0.376 after DOT first‐dose and after non‐DOT strategies, respectively. The corresponding sensitivity were 0.653 and 0.676, with a specificity of 0.70 after DOT first‐dose and after non‐DOT, respectively. The two‐by‐two tables can be viewed in Table 2.

Day 3 cutoff concentrations for any given bodyweight, dose, pretreatment parasite density, and pregnancy status can be derived from the developed R‐script (Appendix S1).

## DISCUSSION

Poor medication adherence results in lower drug concentrations, which could increase the risk of treatment failures and in some diseases contribute to resistance development. The predictive ability of two population‐based PK approaches for adherence assessment were evaluated here. The results suggest that the percentile method and the Bayesian approach were both clinically useful in predicting adherence. In addition, the percentile method performed better than the Bayesian approach when evaluating the adherence to more than one dose events and the predictive ability was highly dependent on sample collection time and the magnitude of IIV.

Population‐based PK approaches to assess the medication adherence are not new and have been studied before.^7^, ^10^ A previous evaluation of the Bayesian approach proposed a methodological solution for adherence assessment, and also investigated the impact of variability.^7^ Chao and colleagues calculated the 1st, 2.5th, and 5th percentile cutoff concentrations of lamivudine at predose and 4 h after doses in 2000 simulated individuals, based on a developed population PK model. They also investigated the sensitivity of the adherence predictions if patients missed the last dose or the two last doses.^10^ A recent study used a population‐based PK approach to assess the adherence of a long half‐life drug, tenofovir diphosphate.^35^ However, additional investigations of the predictive ability are needed before the methods can be utilized in a clinical setting. We expanded these approaches to wider conditions, such as various model structures, different half‐lives, between‐patient variability, and bioanalytical assay limitations. In this study the magnitude of IIV had an impact on the predictive performance for both the percentile method and the Bayesian approach. This might be attributed to the high degree of overlap between the probability density distributions after full and poor adherence, which is consistent with previous findings.^7^ Interestingly, in order to achieve a good predictive performance, samples should be collected relatively soon after a planned dosing event, generally between peak concentrations and one half‐life of the drug in this case. The most probable explanation for these findings is that the maximum absolute difference in concentration will be seen at peak concentrations, and the absolute difference will decrease as concentrations get lower. Additionally, similar predictive performance was observed with a more complicated structural model (a 2‐ and 3‐compartment disposition model). These findings suggested that the choice of structural model might not affect the predictive ability, as the IIV and sampling time are the major factors in adherence assessment with both methods.

According to the present analysis, the overall predictive performance of the percentile method was superior compared to the Bayesian approach in regards to specificity and sensitivity, when investigating the adherence to more than one dose events. This may be because the Bayesian approach calculates and compares the probability of each scenario, and pick the one with the highest probability while the percentile approach calculates the cutoff concentration based on a full adherence scenario. The cutoff concentration in the Bayesian approach to differentiate between full and nonadherence was somehow higher compared to optimal cutoff concentration in the percentile approach. Moreover, it was found that no unique percentile cutoff values existed for the percentile method. The optimal cutoff percentile value, to discriminate between full and poor adherence, was heavily dependent on the level of between‐patient variability and the sampling time. In general, lower concentration percentile cutoff values were favored with a low magnitude of IIV and early sampling times. The simulations were necessary to derive optimal percentiles and sampling times when using the percentile approach for DOT and non‐DOT scenarios.

Additionally, it might be more informative from a clinical perspective to learn what doses that were missed. However, this would be hard to assess in practice because of the nature of the population‐based PK approach (concentration distribution overlapping due to variability). The most suitable methodology to assess this would be the Bayesian approach as it can derive the probability for each dose event.^7^ In a clinical setting of malaria therapy, the first dose is generally administered directly to patients at the time when malaria is diagnosed. The patients are also most motivated to take this medication because they are ill and seeking medical treatment for their symptoms. It is not entirely clear if it is more adverse for the patient to miss the second or third dose, as this has not been studied in detail. However, from a biological point of view, we would assume that it is more advantageous to be treated on days 1 and 3 compared to days 1 and 2, as the parasite lifecycle is 48 h long in *P. falciparum* infections. Thus, it would most likely be an advantage to cover two parasite lifecycles with high drug concentrations (i.e., treatment on days 1 and 3). However, any PK‐based approach would struggle to distinguish between nonadherence on day 2 or day 3, based on a drug measurement taken later in time, and therefore the exact pattern of nonadherence could not be evaluated reliably with the methods presented here.

Varying degrees of complexity of a correctly specified absorption model should have a minimal impact on the elimination phase. More complex absorption models (e.g., parallel or sequential absorption pathways), is therefore unlikely to affect the adherence assessment, because the proposed sampling time is between peak concentrations and one half‐life after dose. Moreover, the impact of flip‐flop kinetics on adherence is expected to be minimal, because the model predictions (i.e., distribution of concentrations at 1 half‐life after dose) will not change. Moreover, model complexity, the amount of underlying data for model development, and possible model misspecification will result in varying degrees of uncertainty in parameter estimates and translate in uncertainty in predicted concentration distributions. This will in turn impact the performance of any PK‐based approach, and add noise to the possibility of identifying nonadherence. This was not evaluated in the developed methodology as it can be minimized by developing parsimonious models, based on informative data.

From a clinical application aspect, a big challenge of the population‐based PK approach is to choose the correct PK model, reflecting the true structural model, parameter distribution, and covariate effects. The choice of model will have a high impact on the result and it is important to use a validated model. Large scale population PK models, based on individual patient data meta‐analyses, should have the best chance to accurately described parameter distributions due to the large and diverse sample size,^36^ and will therefore be optimal to use for adherence assessments. Another issue in the clinical application is how many dose events that can be evaluated, considering the large number of poor compliance scenarios that are possible for therapies with a large number of administered doses. This might result in lower power to identify adherence as well as computationally intensive evaluations. This suggests that any adherence assessments can only really be reliably accomplished for recent doses.

In the current paper, the used population PK models for piperaquine and lumefantrine were developed using data from thousands of patients, accurately reflecting the true distribution of PK parameters. For the percentile method, concentration measurements of piperaquine at day 7 were optimal to assess the adherence for DOT first‐dose and non‐DOT strategies. For lumefantrine, day 3 concentrations can be used for both DOT first‐dose and non‐DOT strategies. The predictive performance for the two antimalarial partner drugs was only fair (AUC under the ROC curve was 0.75) with the optimal sampling time (within 0.5 half‐life), due to the high variability seen in PK parameters. An ideal scenario of low variability (<20%) would achieve excellent predictive performance (AUC under the ROC curve of 0.9, as shown in Figure S1). An approach with a fair overall performance would still be useful in a clinical setting, as demonstrated in previous publication when evaluating adherence to seasonal malaria chemoprevention (SMC), demonstrating that less than 20% of children were fully adherent (PK‐based percentile approach) as compared to 75.8% adherent by self‐report.^37^ These results confirmed the value of the PK‐based adherence approach in a real clinical setting and suggests that efforts are needed to improve the adherence to seasonal malaria chemoprevention among children in this area.

Moreover, the predictive performance for these commonly used antimalarial partner drugs might be improved by combining it with efficacy end points, such as parasitemia on day 28. However, genuine treatment failures might be a result of nonadherence and/or parasite resistance, which makes it difficult to generalize such an approach due to region‐specific factors. Furthermore, distinction between re‐infections and recrudescent infections require genotyping of the parasite and is not practically feasible with *vivax* malaria due to the liver reservoir of hypnozoites.

We also demonstrated that the limit of detection, which is commonly used in adherence assessments for antimalarial therapy, is not an optimal cutoff value as this would result in much lower concentration than those derived from the proposed percentile method. Furthermore, the limit of detection is assay‐specific and highly sensitive liquid‐chromatography tandem mass spectrometry assays can detect concentrations in the ng/mL‐range. Using a low concentration, based on the limit of detection, would result in a very high specificity but a low sensitivity for assessing adherence. In addition, as a general rule, it would be very difficult to evaluate any drug with an assay LOQ limit above what is needed to quantify drug concentrations in the terminal elimination phase. Thus, we believe that sampling within one half‐life of the last dose, should produce a sample that can be quantified reliably for adherence assessments. We also assessed the influence of LOQ on the predictive performance, and demonstrated that this impact was relatively small if the fraction of LOQ data was less than 20%.

One limitation of the current work is that an equiprobable combination of dose events, from full adherence to worst nonadherence, were used in the simulations. This may not fully reflect clinical nonadherence scenarios. An additional limitation is that the PK model must reflect the true PK structure as well as between‐patient variability to derive accurate cutoff values, and this is not always available, especially for drugs used to treat neglected tropical diseases.

## CONCLUSION

The population‐based percentile method performed similar or better for adherence predictions compared to the Bayesian approach in the different dosing strategies evaluated here. Between‐patient PK variability and sample collection time had a large impact on the predictive ability to distinguish nonadherence from full adherence. The percentile method was successfully applied to derive optimal cutoff concentration values for two of the most commonly used antimalarial drugs, when sampled on day 3 (lumefantrine) and day 7 (piperaquine) after treatment initiation.

## AUTHOR CONTRIBUTIONS

J.D., R.M.H., and J.T. wrote the manuscript. J.D. and J.T. designed the research. J.D. analyzed the data.

## FUNDING INFORMATION

This work was supported by the Wellcome Trust (220211).

## CONFLICT OF INTEREST STATEMENT

The authors declared no competing interests for this work.

## CONSENT FOR PUBLICATION

All authors provided consent for publication. For the purpose of open access, the author has applied a CC BY public copyright license to any Author Accepted Manuscript version arising from this submission.

## CODE AVAILABILITY

Code availability is given in Appendix S1.

## Supporting information


Appendix S1

## Data Availability

All data supporting the findings of this study are available within the paper.

## References

[1] Banek K , Lalani M , Staedke SG , Chandramohan D . Adherence to artemisinin‐based combination therapy for the treatment of malaria: a systematic review of the evidence. Malar J. 2014;13:7. 24386988 10.1186/1475-2875-13-7 PMC3893456

[2] Neiman AB , Ruppar T , Ho M , et al. CDC grand rounds: improving medication adherence for chronic disease management – innovations and opportunities. MMWR Morb Mortal Wkly Rep. 2017;66:1248‐1251. 29145353 10.15585/mmwr.mm6645a2 PMC5726246

[3] Osterberg L , Blaschke T . Adherence to medication. N Engl J Med. 2005;353:487‐497. 16079372 10.1056/NEJMra050100

[4] Bruxvoort K , Goodman C , Kachur SP , Schellenberg D . How patients take malaria treatment: a systematic review of the literature on adherence to antimalarial drugs. PLoS One. 2014;9:e84555. 24465418 10.1371/journal.pone.0084555 PMC3896377

[5] Alemayehu C , Mitchell G , Nikles J . Barriers for conducting clinical trials in developing countries – a systematic review. Int J Equity Health. 2018;17:37. 29566721 10.1186/s12939-018-0748-6 PMC5863824

[6] Bruxvoort K , Festo C , Cairns M , et al. Measuring patient adherence to malaria treatment: a comparison of results from self‐report and a customised electronic monitoring device. PLoS One. 2015;10:e0134275. 26214848 10.1371/journal.pone.0134275 PMC4516331

[7] Barriere O , Li J , Nekka F . A Bayesian approach for the estimation of patient compliance based on the last sampling information. J Pharmacokinet Pharmacodyn. 2011;38:333‐351. 21445612 10.1007/s10928-011-9196-2

[8] Knights J , Rohatagi S . Development and application of an aggregate adherence metric derived from population pharmacokinetics to inform clinical trial enrichment. J Pharmacokinet Pharmacodyn. 2015;42:263‐273. 25821065 10.1007/s10928-015-9414-4 PMC4432109

[9] Tu W , Nyandiko WM , Liu H , et al. Pharmacokinetics‐based adherence measures for antiretroviral therapy in HIV‐infected Kenyan children. J Int AIDS Soc. 2017;20:21157. 28605170 10.7448/IAS.20.1.21157 PMC5515048

[10] Zhang C , Denti P , van der Walt J‐S , et al. Population pharmacokinetic model for adherence evaluation using lamivudine concentration monitoring. Ther Drug Monit. 2012;34:481‐484. 22722777 10.1097/FTD.0b013e31825c6067

[11] World Health Organization . World Malaria Report . 2022.

[12] World Health Organization . Guidelines for the Treatment of Malaria . 2015.

[13] Douglas NM , Poespoprodjo JR , Patriani D , et al. Unsupervised primaquine for the treatment of *Plasmodium vivax* malaria relapses in southern Papua: a hospital‐based cohort study. PLoS Med. 2017;14:e1002379. 28850568 10.1371/journal.pmed.1002379 PMC5574534

[14] Bell DJ , Wootton D , Mukaka M , et al. Measurement of adherence, drug concentrations and the effectiveness of artemether‐lumefantrine, chlorproguanil‐dapsone or sulphadoxine‐pyrimethamine in the treatment of uncomplicated malaria in Malawi. Malar J. 2009;8:204. 19709418 10.1186/1475-2875-8-204 PMC2744923

[15] Faucher JF , Aubouy A , Adeothy A , et al. Comparison of sulfadoxine‐pyrimethamine, unsupervised artemether‐lumefantrine, and unsupervised artesunate‐amodiaquine fixed‐dose formulation for uncomplicated *Plasmodium falciparum* malaria in Benin: a randomized effectiveness noninferiority trial. J Infect Dis. 2009;200:57‐65. 19469703 10.1086/599378

[16] Fogg C , Bajunirwe F , Piola P , et al. Adherence to a six‐dose regimen of artemether‐lumefantrine for treatment of uncomplicated *Plasmodium falciparum* malaria in Uganda. Am J Trop Med Hyg. 2004;71:525‐530. 15569777

[17] Piola P , Fogg C , Bajunirwe F , et al. Supervised versus unsupervised intake of six‐dose artemether‐lumefantrine for treatment of acute, uncomplicated *Plasmodium falciparum* malaria in Mbarara, Uganda: a randomised trial. Lancet. 2005;365:1467‐1473. 15850630 10.1016/S0140-6736(05)66416-1

[18] Rahman MM , Dondorp AM , Day NPJ , et al. Adherence and efficacy of supervised versus non‐supervised treatment with artemether/lumefantrine for the treatment of uncomplicated *Plasmodium falciparum* malaria in Bangladesh: a randomised controlled trial. Trans R Soc Trop Med Hyg. 2008;102:861‐867. 18606428 10.1016/j.trstmh.2008.05.022

[19] Simba DO , Kakoko D , Tomson G , et al. Adherence to artemether/lumefantrine treatment in children under real‐life situations in rural Tanzania. Trans R Soc Trop Med Hyg. 2012;106:3‐9. 22099005 10.1016/j.trstmh.2011.09.006

[20] Congpuong K , Bualombai P , Banmairuroi V , Na‐Bangchang K . Compliance with a three‐day course of artesunate‐mefloquine combination and baseline anti‐malarial treatment in an area of Thailand with highly multidrug resistant falciparum malaria. Malar J. 2010;9:43. 20132537 10.1186/1475-2875-9-43 PMC2829592

[21] Shwe T , Lwin M , Aung S . Influence of blister packaging on the efficacy of artesunate + mefloquine over artesunate alone in community‐based treatment of non‐severe falciparum malaria in Myanmar. Bull World Health Organ. 1998;76(Suppl 1):35‐41. 9763721 PMC2305574

[22] Bigira V , Kapisi J , Clark TD , et al. Protective efficacy and safety of three antimalarial regimens for the prevention of malaria in young Ugandan children: a randomized controlled trial. PLoS Med. 2014;11:e1001689. 25093754 10.1371/journal.pmed.1001689 PMC4122345

[23] Na‐Bangchang K , Congpuong K , Sirichaisinthop J , Suprakorb K , Karbwang J . Compliance with a 2 day course of artemether‐mefloquine in an area of highly multi‐drug resistant *Plasmodium falciparum* malaria. Br J Clin Pharmacol. 1997;43:639‐642. 9205825 10.1046/j.1365-2125.1997.00604.x PMC2042779

[24] Hoglund RM , Workman L , Edstein MD , et al. Population pharmacokinetic properties of piperaquine in falciparum malaria: an individual participant data meta‐analysis. PLoS Med. 2017;14:e1002212. 28072872 10.1371/journal.pmed.1002212 PMC5224788

[25] Kloprogge F , Workman L , Borrmann S , et al. Artemether‐lumefantrine dosing for malaria treatment in young children and pregnant women: a pharmacokinetic‐pharmacodynamic meta‐analysis. PLoS Med. 2018;15:e1002579. 29894518 10.1371/journal.pmed.1002579 PMC5997317

[26] Cohen J , Saran I . The impact of packaging and messaging on adherence to malaria treatment: evidence from a randomized controlled trial in Uganda. J Dev Econ. 2018;134:68‐95. 30177864 10.1016/j.jdeveco.2018.04.008 PMC6088513

[27] Youden WJ . Index for rating diagnostic tests. Cancer. 1950;3:32‐35. 15405679 10.1002/1097-0142(1950)3:1<32::aid-cncr2820030106>3.0.co;2-3

[28] Hosmer D . Applid Logistic Regression. John Wiley and Sons; 2000:160‐164.

[29] Fan J , Upadhye S , Worster A . Understanding receiver operating characteristic (ROC) curves. CJEM. 2006;8:19‐20. 17175625 10.1017/s1481803500013336

[30] Tarning J , Lindegårdh N , Annerberg A , et al. Pitfalls in estimating piperaquine elimination. Antimicrob Agents Chemother. 2005;49:5127‐5128. 16304183 10.1128/AAC.49.12.5127-5128.2005 PMC1315981

[31] WorldWide Antimalarial Resistance Network Lumefantrine PKPDSG . Artemether‐lumefantrine treatment of uncomplicated *Plasmodium falciparum* malaria: a systematic review and meta‐analysis of day 7 lumefantrine concentrations and therapeutic response using individual patient data. BMC Med. 2015;13:227. 26381375 10.1186/s12916-015-0456-7 PMC4574542

[32] Price RN , Uhlemann AC , van Vugt M , et al. Molecular and pharmacological determinants of the therapeutic response to artemether‐lumefantrine in multidrug‐resistant *Plasmodium falciparum* malaria. Clin Infect Dis. 2006;42:1570‐1577. 16652314 10.1086/503423 PMC4337983

[33] Price RN , Hasugian AR , Ratcliff A , et al. Clinical and pharmacological determinants of the therapeutic response to dihydroartemisinin‐piperaquine for drug‐resistant malaria. Antimicrob Agents Chemother. 2007;51:4090‐4097. 17846129 10.1128/AAC.00486-07 PMC2151469

[34] Tarning J , Zongo I , Somé FA , et al. Population pharmacokinetics and pharmacodynamics of piperaquine in children with uncomplicated falciparum malaria. Clin Pharmacol Ther. 2012;91:497‐505. 22258469 10.1038/clpt.2011.254 PMC3736305

[35] Devanathan AS , Dumond JB , Anderson DJC , et al. A novel algorithm to improve PrEP adherence monitoring using dried blood spots. Clin Pharmacol Ther. 2023;113:896‐903. 36622798 10.1002/cpt.2845 PMC10023501

[36] He C , Griffies A , Liu X , Adamczyk R , Huang SP . A pooled analysis of pharmacokinetic variability information for common probe substrates used in drug‐drug interaction studies. Pharmacology. 2018;101:170‐175. 29402842 10.1159/000485516

[37] Ding J , Coldiron ME , Assao B , et al. Adherence and population pharmacokinetic properties of amodiaquine when used for seasonal malaria chemoprevention in African children. Clin Pharmacol Ther. 2020;107:1179‐1188. 31652336 10.1002/cpt.1707 PMC7232861

