# Supplementary material for: Medication adherence framework: A population‐based pharmacokinetic approach and its application in antimalarial treatment assessments
Source: CPT Pharmacometrics Syst Pharmacol. 2024 Mar 25;13(5):795–811. doi: 10.1002/psp4.13119 (PMC11098161; doi:10.1002/psp4.13119)
Supplement: Supplementary file 1 — Appendix S1 [file PSP4-13-795-s001.docx]

**Supplementary material**

**Medication Adherence Framework: A Population-based Pharmacokinetic Approach
and Its Application in Antimalarial Treatment Assessments**

*Junjie Ding, Richard M. Hoglund, Joel Tarning*

**Figure S1.** The Youden’s index (left column) of the percentile method and the Bayesian approach and receiver operating characteristic (ROC) curve (right curve) derived from the percentile method at different levels of inter-individual variability, at different sampling times after the last dose. Adherence to the last 2 doses (DOT one-dose) was evaluated in comparison to patients with complete adherence of receiving a daily dose for 3 days. The Youden index was derived for each time point (every 1 hour) up to 72 hours after the last dose.

**Figure S2.** The Youden’s index (left column) of the percentile method and the Bayesian approach and receiver operating characteristic (ROC) curve (right column) derived from the percentile method based on a 2-compartment disposition model, at different sampling time after the last dose. The different adherence scenarios were evaluated in comparison to patients with complete adherence of receiving a daily dose for 3 days. The Youden index was derived for each time point (every 1 hour) up to 72 hours after the last dose. DOT: directly observed therapy.

**Figure S3.** The Youden’s index (left column) of the percentile method and the Bayesian approach and receiver operating characteristic (ROC) curve (right column) derived from the percentile method based on a 3-compartment disposition model, at different sampling time after the last dose. The different adherence scenarios were evaluated in comparison to patients with complete adherence of receiving a daily dose for 3 days. The Youden index was derived for each time point (every 1 hour) up to 72 hours after the last dose.DOT: directly observed therapy.

**Figure S4.** The Youden’s index (left column) of the percentile method and the Bayesian approach and receiver operating characteristic (ROC) curve (right column) derived from the percentile method based on a drug with a shorter half-life of 8 hours, at different sampling time after the last dose. The different adherence scenarios were evaluated in comparison to patients with complete adherence of receiving a daily dose for 3 days. The Youden index was derived for each time point (every 1 hour) up to 72 hours after the last dose.DOT: directly observed therapy.

**Figure S5.** Receiver operating characteristic (ROC) curve derived from the percentile method based on a 1-compartment disposition model, at 24 hours sampling time after the last dose, evaluating the performance at different fractions of censored data.

**Table S1**. 2×2 table to derive diagnostic parameters for adherence assessment.

**R SCRIPTS**

R-script for calculation of the day 7 cut-off concentration for piperaquine in children weighting less than 11 kg.

R-script for calculation of the day 3 cut-off concentration for lumefantrine.


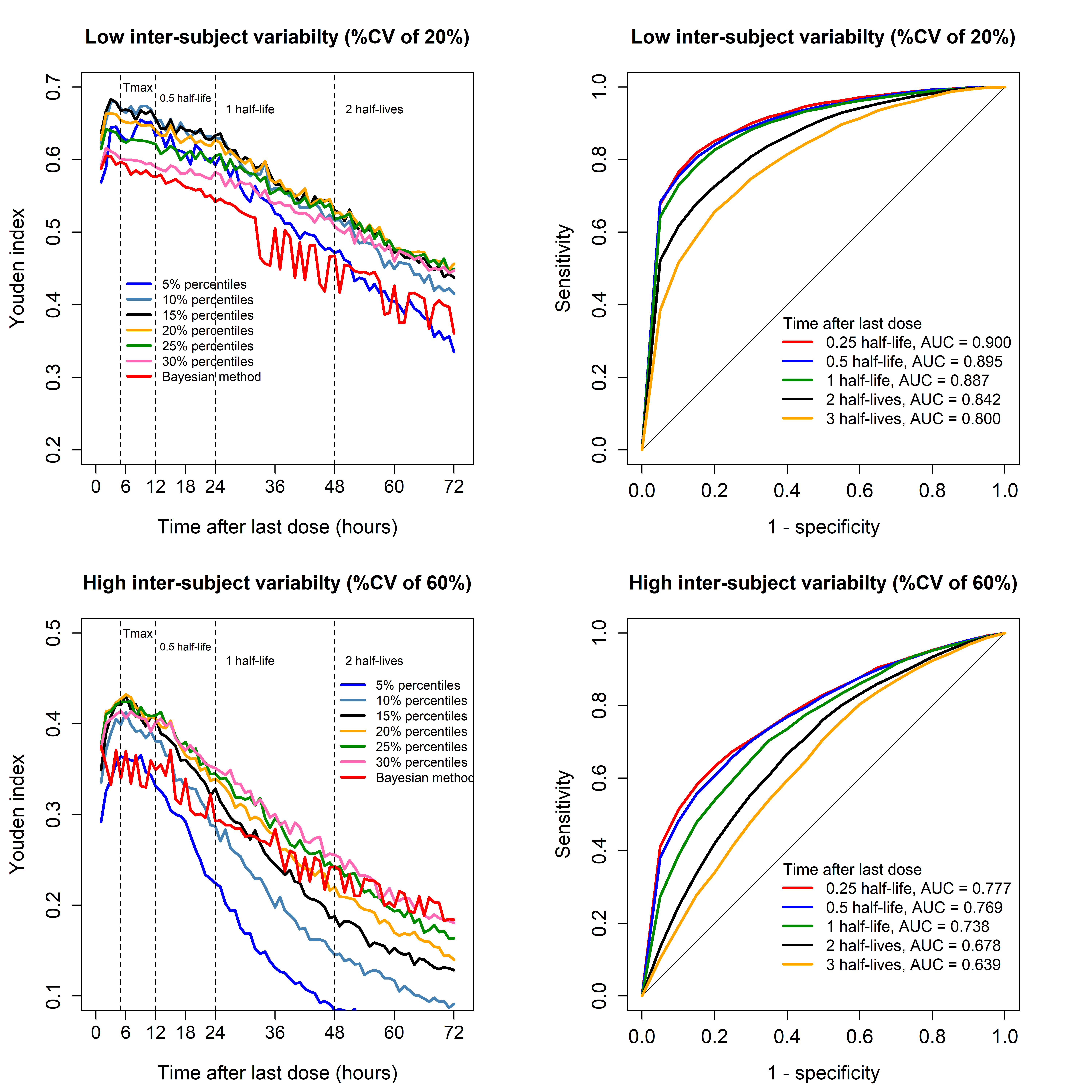


**Figure S1.** The Youden’s index (left panel) of the percentile method and the Bayesian approach and receiver operating characteristic (ROC) curve (right panel) derived from the percentile method at different levels of inter-individual variability, at different sampling times after the last dose. Adherence to the last 2 doses (DOT one-dose) was evaluated in comparison to patients with complete adherence of receiving a daily dose for 3 days. The Youden index was derived for each time point (every 1 hour) up to 72 hours after the last dose.


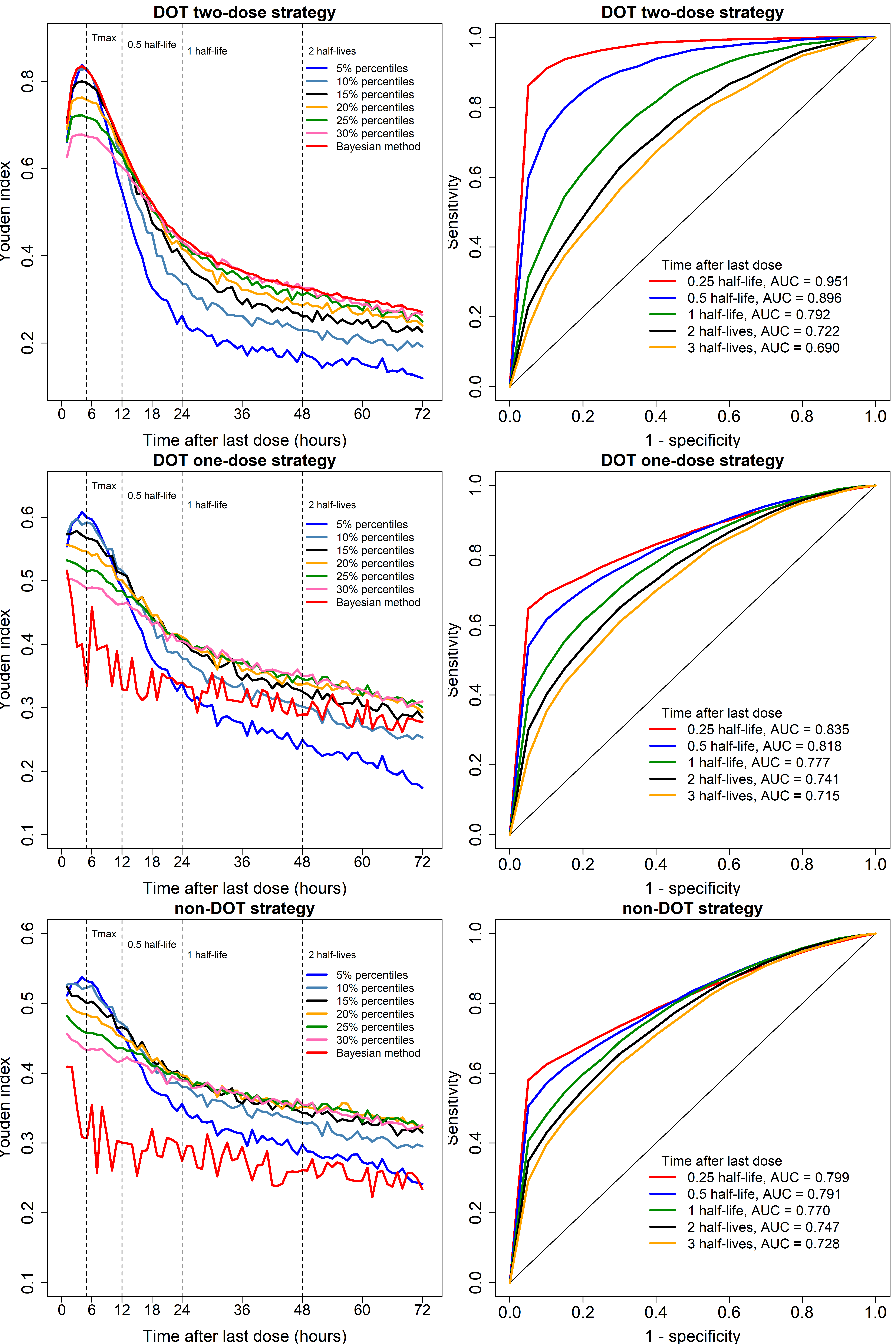


**Figure S2.** The Youden’s index (left panel) of the percentile method and the Bayesian approach and receiver operating characteristic (ROC) curve (right panel) derived from the percentile method based on a 2-compartment disposition model, at different sampling times after the last dose. The different adherence scenarios were evaluated in comparison to patients with complete adherence of receiving a daily dose for 3 days. DOT: directly observed therapy. The Youden index was derived for each time point (every 1 hour) up to 72 hours after the last dose.

**
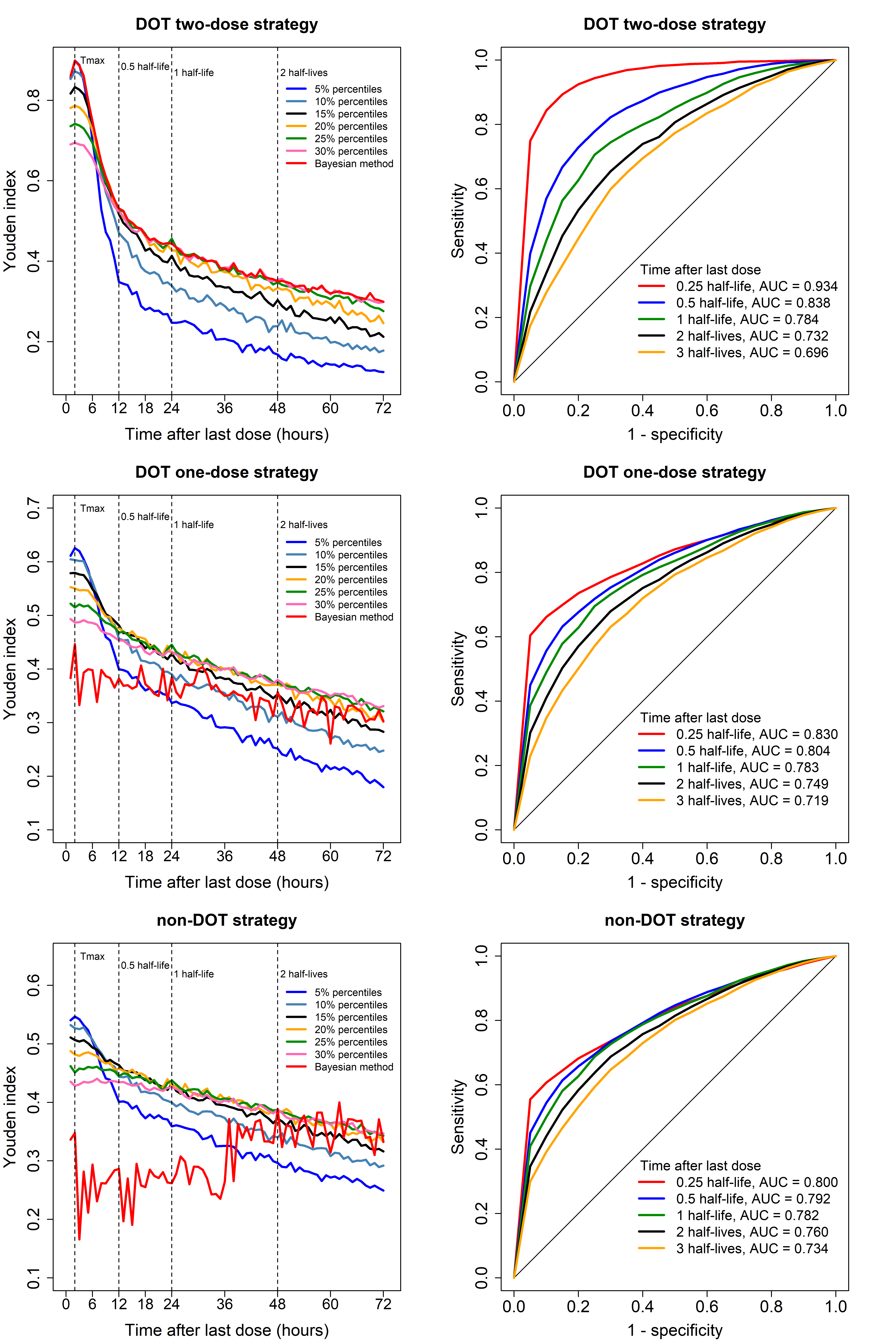
**

**Figure S3.** The Youden’s index (left panel) of the percentile method and the Bayesian approach and receiver operating characteristic (ROC) curve (right panel) derived from the percentile method based on a 3-compartment disposition model, at different sampling times after the last dose. The different adherence scenarios were evaluated in comparison to patients with complete adherence of receiving a daily dose for 3 days. DOT: directly observed therapy. The Youden index was derived for each time point (every 1 hour) up to 72 hours after the last dose.

**
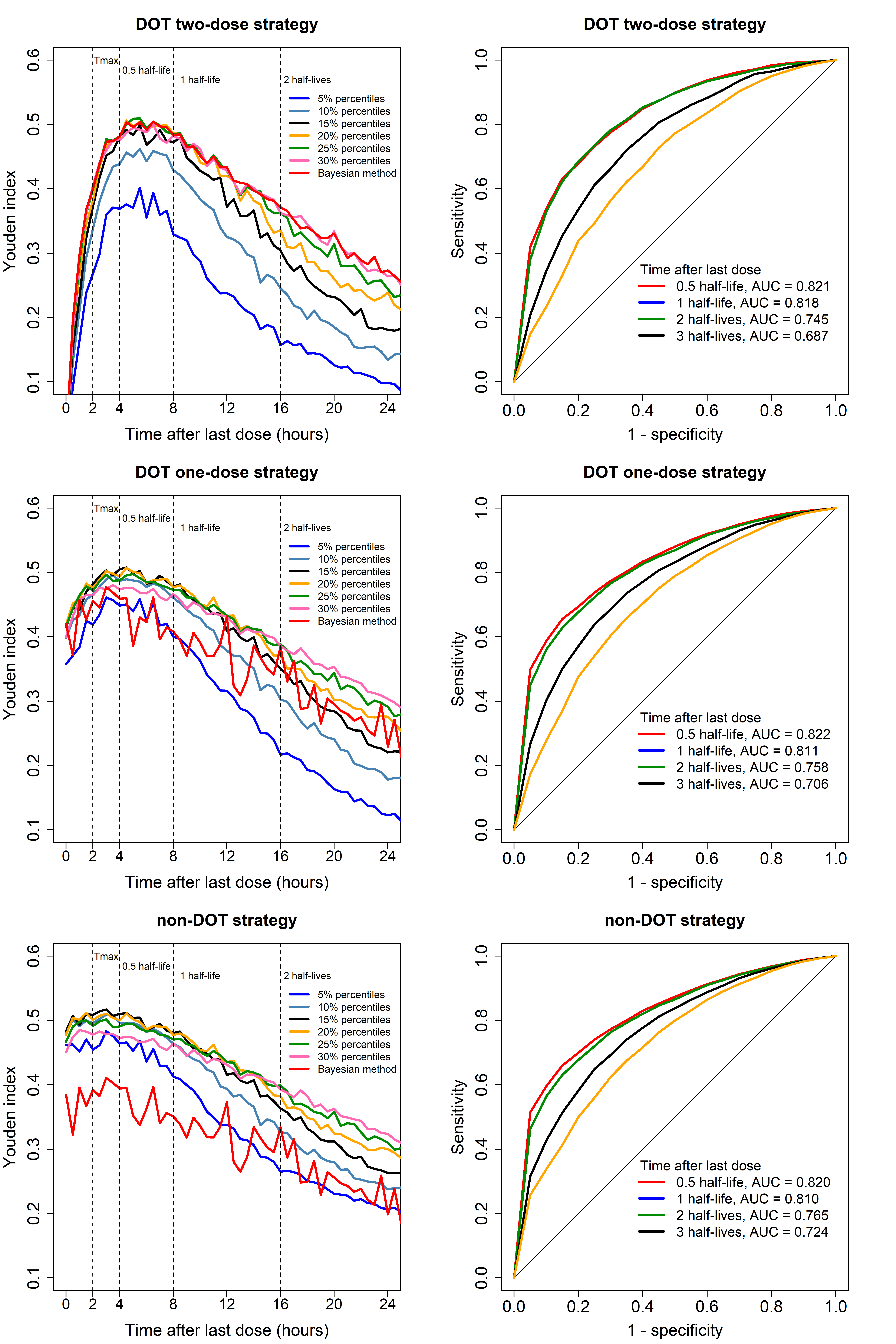
**

**Figure S4.** The Youden’s index (left panel) of the percentile method and the Bayesian approach and receiver operating characteristic (ROC) curve (right panel) derived from the percentile method based on a drug with a shorter half-life of 8 hours, at different sampling times after the last dose. The different adherence scenarios were evaluated in comparison to patients with complete adherence of receiving a daily dose for 3 days. DOT: directly observed therapy. The Youden index was derived for each time point (every 1 hour) up to 24 hours after the last dose.

**
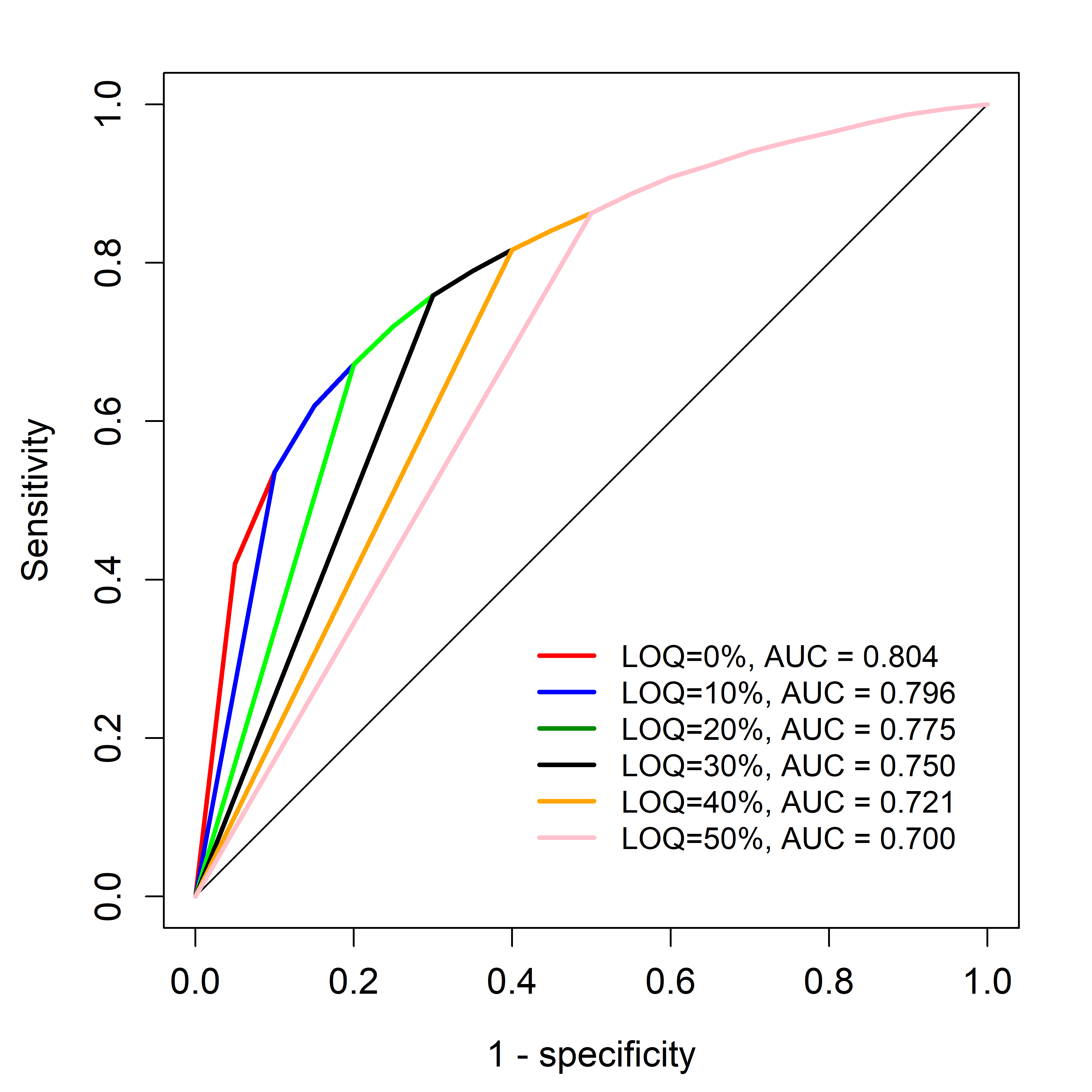
**

**Figure S5.** Receiver operating characteristic (ROC) curve derived from the percentile method based on a 1-compartment disposition model and a sample collected at 1 half-life (i.e., 24 hours) after the last dose, evaluating the predictive performance at different fractions of censored data.

**Table S1**. 2×2 table to derive diagnostic parameters for adherence assessment.

|  | Simulated adherence | |
| --- | --- | --- |
| PK-based approach assessed adherence | Non-adherence | Full adherence |
| Non-adherence | TP | FP |
| Full-adherence | FN | TN |
| Total | TP +FN =2,000 (DOT 2-dose) or 6,000 (DOT 1-dose) or 14,000 (non-DOT) | FP + TN = 2,000 |

DOT: directly observed therapy; TP: true-positive, TN: true-negative, FP: false-positive, FN: false-negative; Sensitive = TP/(TP + FN); Specificity = TN/(TN + FP).

**R-script for calculating the day 7 cut-off concentrations for piperaquine in children weighting less than 11 kg.**

## Load R package

library(deSolve)

## Define PK model (compartments and differential equation)

pkmodel=function(time,y,parms){

depot=y[1] # Gut compartments

centr1=y[2] # Central compartment

peri1=y[3] # Peripheral compartment 1

peri2=y[4] # Peripheral compartment 2

delay1=y[5] # Transit compartment 1

delay2=y[6] # Transit compartment 2

# Differential equations

y=c()

with(as.list(c(y, parms)), {

C2 = centr1/V2;

C3 = peri1/V3;

C4 = peri2/V4

NN = 2

KTR = (NN+1)/MTT

y[1] = - KTR*depot

y[2] = KTR*delay2 - CL1*C2 - Q1*C2 + Q1*C3 - Q2*C2 + Q2*C4

y[3] = Q1*C2 - Q1*C3

y[4] = Q2*C2 - Q2*C4

y[5] = KTR * depot - KTR * delay1

y[6] = KTR * delay1 - KTR * delay2

list(c(y))

})}

# Initial value for each compartment

yini=c(depot = 0, centr1 = 0, peri1 = 0, peri2 = 0, delay1 = 0, delay2 = 0)

times <- seq(0,672, by = 1) #sampling up to day 28 (672 hours) after first dose

wt <- 8 # bodyweight in kg

if (wt>=8 & wt <11) { #dose amount (mg) for individual weighted <10 kg

dose = 240 * 0.577

} else {

dose = 160 * 0.577

}

age <- 0.6 #age in years

## Simulation

nsub = 2000 # Numbers of simulated individuals

set.seed(20180530)

allsim111<-data.frame()

# Input parameters and their variability

for(i in 1:nsub){

parameters <- cbind(MTT = 2.11*exp(rnorm(1, 0, 0.38) + rnorm(1,0,0.464)), CL1 = 55.4*((wt/54)**0.75)*(age**5.51/(0.575**5.51+age**5.51))*exp(rnorm(1, 0, 0.279)),

V2 = 2910*(wt/54)*exp(rnorm(1, 0, 0.67)),F1 = 1*exp(rnorm(1, 0, 0.414) + rnorm(1,0,0.535)),Q1 = 310*((wt/54)**0.75),Q2 = 105*((wt/54)**0.75)*exp(rnorm(1, 0, 0.236)),

V3 = 4910*(wt/54)*exp(rnorm(1, 0, 0.24)),V4 = 30900*(wt/54)*exp(rnorm(1, 0, 0.347)))

parms=parameters[1,]

# Dose regimens

events=data.frame(var="depot",time=c(0,24,48),value=c(parameters[,"F1"][1]*dose,parameters[,"F1"][1]*dose*1.237,parameters[,"F1"][1]*dose*1.474),method="add")

# Perform simulation and output results

out <- lsoda(yini, times=times, pkmodel, parms=parameters[1,], events=list(data=events))

sim111=data.frame(id=i,parameters,as.data.frame(out))

allsim111<-rbind(allsim111, sim111)

}

# Rename column of simulation output

colnames(allsim111) <- c('id',"ktr","cl1","v2","f1","q1",'q2',"v3",'v4',"time","A1", "A2",'A3','A4','A5','A6')

# Incorporate residue error

allsim111$ipred <- (1000*allsim111$A2/allsim111$v2) * (1 + (rnorm(length(allsim111$A2), 0, 0.339)))

## Output cut-off concentration at a given time and a pre-defined percentile cut-off value

stime <- 168 # Day 7 samples

allsim111_st <- subset(allsim111,time==stime)

cutoff20<-as.numeric(quantile(allsim111_st$ipred, 0.20)) # 20% percentile cut-off

**R-script for calculating the day 3 cut-off concentration for lumefantrine.**

## Load R package

library(deSolve)

## Define PK model (compartments and differential equation)

pkmodel=function(time,y,parms){

depot=y[1] # Gut compartments

centr1=y[2] # Central compartment

peri1=y[3] # Peripheral compartment

# Differential equations

y=c()

with(as.list(c(y, parms)), {

C2 = centr1/V2;

C3 = peri1/V3;

y[1] = - ka*depot

y[2] = ka*depot - CL1*C2 - Q1*C2 + Q1*C3

y[3] = Q1*C2 - Q1*C3

list(c(y))

})}

# Initial value for each compartment

yini=c(depot = 0, centr1 = 0, peri1 = 0)

times <- seq(0,504, by = 1) # Sampling up to day 21 (504 hours) after first dose

dose <- 480 ## 120 mg per tablet, a total of 4 tablets were administered

wt <- 42 # Bodyweight (kg) of a given patient

preg <- 0 # Pregnant status, 1 and 0 represent pregnancy and non-pregnancy

dosewt <- dose/wt ## dose in mg/kg

fdose <- 1 - (dosewt/(3.86+dosewt)) # Saturation effect of dose on relative bioavailability

para <- 15800 # Baseline parasite density (/μL)

logpara <- log(para)

fpara <- (logpara/4.2)**-0.643 # Effect of baseline parasite density on relative bioavailability

## Simulation

allsim <- data.frame()

nsub = 2000 # Numbers of simulated individuals

set.seed(201806)

# Input parameters and their variability

for(i in 1:nsub){

parameters <- cbind(ka = 0.0386 * (1+0.352*preg), CL1 = 1.35*((wt/42)**0.75), V2 = 11.2*(wt/42)*exp(rnorm(1, 0, 1.06)), F1 = 1*fdose*fpara*exp(((exp(rnorm(1, 0, 0.634))**-0.343-1)/-0.343)),Q1 = 0.344*((wt/42)**0.75),V3 = 59.0*(wt/42))

parms=parameters[1,]

# Dose regimens

events=data.frame(var="depot",time=c(0,8,24,36,48,60),value=c(parameters[,"F1"][1]*dose)ssss,method="add")

# Perform simulation and output results

out <- lsoda(yini, times=times, pkmodel, parms=parameters[1,], events=list(data=events))

sim=data.frame(id=i,parameters,as.data.frame(out))

allsim <-rbind(allsim, sim)

}

# Rename column of simulation output

colnames(allsim) <- c('id',"ka","cl1","v2","f1","q1","v3","time","A1","A2",'A3')

# Incorporate residue error

allsim$ipred <- (1000*allsim$A2/allsim$v2) * exp((rnorm(length(allsim$A2), 0, 0.323)))

## Output cut-off concentration at a given time and a pre-defined percentile cut-off value

stime <- 72 # Day 3 samples, if using day 7 samples, stime need change to 168

allsim_st <- subset(allsim,time==stime)

cutoff30<-as.numeric(quantile(allsim_st$ipred, 0.30)) # 30% percentile
